# A Bayesian Network Decision Support Tool for Low Back Pain Using a RAND Appropriateness Procedure: Proposal and Internal Pilot Study

**DOI:** 10.2196/21804

**Published:** 2021-01-15

**Authors:** Adele Hill, Christopher H Joyner, Chloe Keith-Jopp, Barbaros Yet, Ceren Tuncer Sakar, William Marsh, Dylan Morrissey

**Affiliations:** 1 Sport and Exercise Medicine Queen Mary University of London London United Kingdom; 2 Electronics, Engineering and Computer Science Queen Mary University of London London United Kingdom; 3 Barts Health NHS Trust London United Kingdom; 4 Graduate School of Informatics Middle East Technical University Ankara Turkey; 5 Department of Industrial Engineering Hacettepe University Ankara Turkey

**Keywords:** back pain, decision making, Bayesian methods, consensus

## Abstract

**Background:**

Low back pain (LBP) is an increasingly burdensome condition for patients and health professionals alike, with consistent demonstration of increasing persistent pain and disability. Previous decision support tools for LBP management have focused on a subset of factors owing to time constraints and ease of use for the clinician. With the explosion of interest in machine learning tools and the commitment from Western governments to introduce this technology, there are opportunities to develop intelligent decision support tools. We will do this for LBP using a Bayesian network, which will entail constructing a clinical reasoning model elicited from experts.

**Objective:**

This paper proposes a method for conducting a modified RAND appropriateness procedure to elicit the knowledge required to construct a Bayesian network from a group of domain experts in LBP, and reports the lessons learned from the internal pilot of the procedure.

**Methods:**

We propose to recruit expert clinicians with a special interest in LBP from across a range of medical specialties, such as orthopedics, rheumatology, and sports medicine. The procedure will consist of four stages. Stage 1 is an online elicitation of variables to be considered by the model, followed by a face-to-face workshop. Stage 2 is an online elicitation of the structure of the model, followed by a face-to-face workshop. Stage 3 consists of an online phase to elicit probabilities to populate the Bayesian network. Stage 4 is a rudimentary validation of the Bayesian network.

**Results:**

Ethical approval has been obtained from the Research Ethics Committee at Queen Mary University of London. An internal pilot of the procedure has been run with clinical colleagues from the research team. This showed that an alternating process of three remote activities and two in-person meetings was required to complete the elicitation without overburdening participants. Lessons learned have included the need for a bespoke online elicitation tool to run between face-to-face meetings and for careful operational definition of descriptive terms, even if widely clinically used. Further, tools are required to remotely deliver training about self-identification of various forms of cognitive bias and explain the underlying principles of a Bayesian network. The use of the internal pilot was recognized as being a methodological necessity.

**Conclusions:**

We have proposed a method to construct Bayesian networks that are representative of expert clinical reasoning for a musculoskeletal condition in this case. We have tested the method with an internal pilot to refine the process prior to deployment, which indicates the process can be successful. The internal pilot has also revealed the software support requirements for the elicitation process to model clinical reasoning for a range of conditions.

**International Registered Report Identifier (IRRID):**

DERR1-10.2196/21804

## Introduction

Back pain is a prime example of the general increase in long-term musculoskeletal conditions. It has been deemed a leading cause of years lived with disability worldwide, and health care costs for treating back pain are escalating [1]. Some low back pain (LBP) cases are associated with injuries that will self-resolve, but there are a considerable number of people who live with disabling LBP. It is difficult to predict who will have a favorable outcome and who will not. Meta-analyses have consistently shown that treatment outcomes for musculoskeletal conditions are partial [2]. Studies have shown that clinicians may not recognize complexity when assessing these patients [3], and efforts made to improve diagnosis via pattern recognition or analytical approaches [4] have limited success. An artificial intelligence (AI) decision support tool that overcomes one or more of these issues would greatly improve the referral of LBP patients to the correct treatment care pathway, and the approach would be generalizable to other musculoskeletal conditions.

The potential of AI systems to address some of these issues has been recognized by The Digital Framework for Allied Health Professionals in the United Kingdom [5], which highlights the priority to develop digitally mature systems, improve outcomes, and limit variation in service delivery. Similarly, in 2019, Her Majesty’s Government announced a £250 million investment in AI development in the UK National Health Service [6] for such purposes. However, many of these techniques are data driven and thus require large error-free data sets that must, crucially, also contain outcome results. Unfortunately, while there is an abundance of data within the musculoskeletal health care system, access is severely restricted and most of the data are unrefined, often being free text or hand written without the necessary outcomes. Furthermore, studies have found that clinicians are skeptical about the ability of AI to perform at the human level [7], and patient and public involvement forums have highlighted a mistrust of black box systems. Successful adoption of AI therefore requires combining clinical evidence, patient data, and expert opinion to mirror the clinical reasoning process and provide transparency regarding how predictions have been reached.

An LBP decision support tool to guide the treatment of patients has been developed with an expert-opinion Delphi-consensus approach in the Netherlands [8], with some success, although there was a suggestion that the results could be further improved with AI and machine learning techniques [9]. The previous authors particularly highlight the limitations of traditional multivariate regression models and suggest that a Bayesian network (BN) would take into account more clinical aspects of assessment. A BN is a probabilistic model that offers an ideal approach for modeling clinical reasoning as it is capable of combining expert opinion with available evidence to provide probabilistic outcomes for a given scenario [10]. These outcomes can be continuously updated to ensure the most recent clinical knowledge is being used [11] and provide explanations of the predictions arrived at [12].

In order to support clinicians in their decision making, we envisage a BN that can predict a patient’s response to a given treatment or course of action when they first present with LBP (ie, in a primary care setting). In order to do this, we must first characterize the types of LBP possible, and the associated risk factors, signs, and symptoms. We propose eliciting a BN that predicts the probability of a patient having a certain LBP-related presentation and, subsequently, how likely they are to improve in response to a course of action. To be clear, this focus on characterization rather than diagnosis was deliberately taken owing to the lack of accurate diagnostic labels for the majority of people with LBP [13], but the BN was designed to identify patients with serious diagnoses, such as nerve root pain and cauda equina syndrome, where these can be identified. For reasons mentioned above, the clinical knowledge base of our BN must be elicited from experts [14,15]. We also believe that it is important not to constrain the type of LBP considered but use expert opinion to guide our focus. In this paper, we outline a protocol for a BN elicitation process that aims to balance the construction of a complex expert-driven model for the treatment of LBP while minimizing the elicitation burden placed on participants. We also describe a pilot study of this process, which highlights the subsequent results that informed the main protocol, discuss the overall methodology, and consider future implementation of the AI tool.

## Methods

### Design

A BN is comprised of the following three components: (1) variables, quantities of interest, such as age, BMI, and the presence/absence of a condition; (2) structure, the dependence of variables on each other, for instance, the condition may be more prevalent in the elderly but the BMI makes no difference; (3) probabilities, quantification of the structure, for example, the probability of having a condition given the person is of a certain age. 

**Figure 1:**
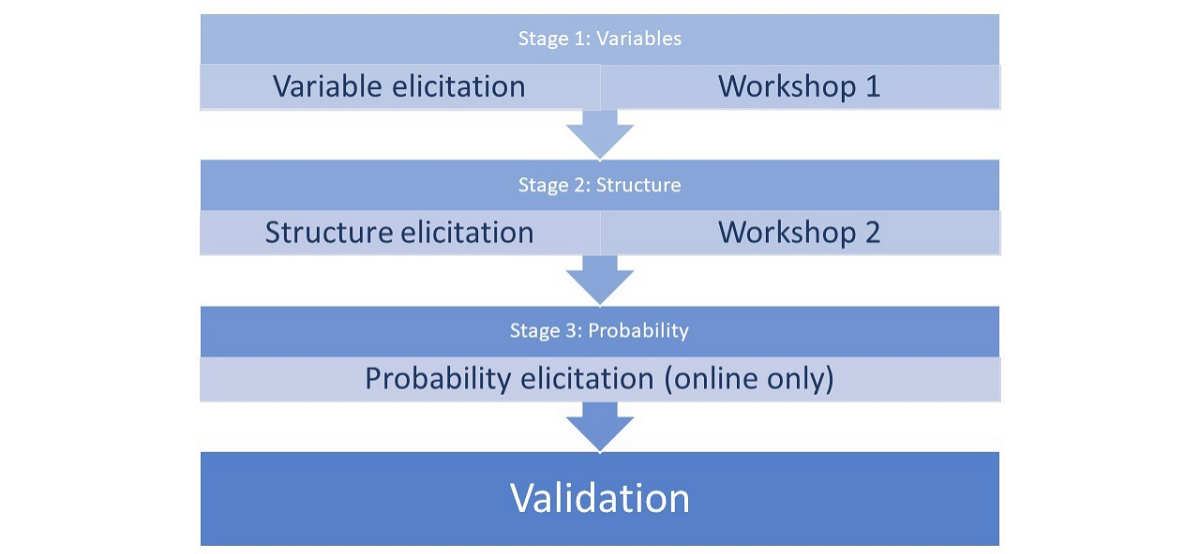
Study procedure.

We therefore divide the elicitation process into three distinct stages related to those components plus a final stage intended as a rudimentary validation of the output from the process (Figure 1).

The overall elicitation method will be a combined Delphi and RAND appropriateness procedure [10]. Participants will first answer questions individually online before contentious results are discussed together in a face-to-face workshop. We have chosen this strategy because related methods have been used with success in previous musculoskeletal studies [8,16-18], and it combines individual tasks and group discussions to explore biases [19]. We acknowledge that further rounds of discussion may help with consensus, but time constraints render this infeasible.

Ethical approval was sought from the Queen Mary University of London, and approval was granted by the Queen Mary Ethics of Research Committee (reference: QMREC2018/48027).

### Participants and Recruitment

Clinicians will be recruited via clinical networks, word of mouth, and social media. There is no definitive number of participants recommended for this style of study. However, for face-to-face workshops, an optimum number of attendees has been suggested to lie between seven and 15 [19]. This number of participants is thought to represent a range of views and promote discussion, without becoming too onerous to manage and facilitate in a workshop setting. We will include extended scope/advanced practitioner physiotherapists (covering orthopedics, rheumatology, neurosurgery, and general musculoskeletal triage services), general practitioners with a special interest in musculoskeletal conditions, and sports medicine doctors. We will exclude clinicians who do not regularly manage patients with LBP or do not hold current professional registration. Participants will be provided with information about the research and be asked to provide informed written consent prior to taking part.

### Data Management

Participants will not be identified in any reports or publications of the results. Any information held by the project team in relation to participants will be kept confidential and managed in accordance with the Data Protection Act, the General Data Protection Regulation (GDPR), the UK Policy Framework for Health and Social Care Research, and the conditions of the Research Ethics Committee.

### Elicitation Process

#### Stage 1: Variable Elicitation

**Figure 2:**
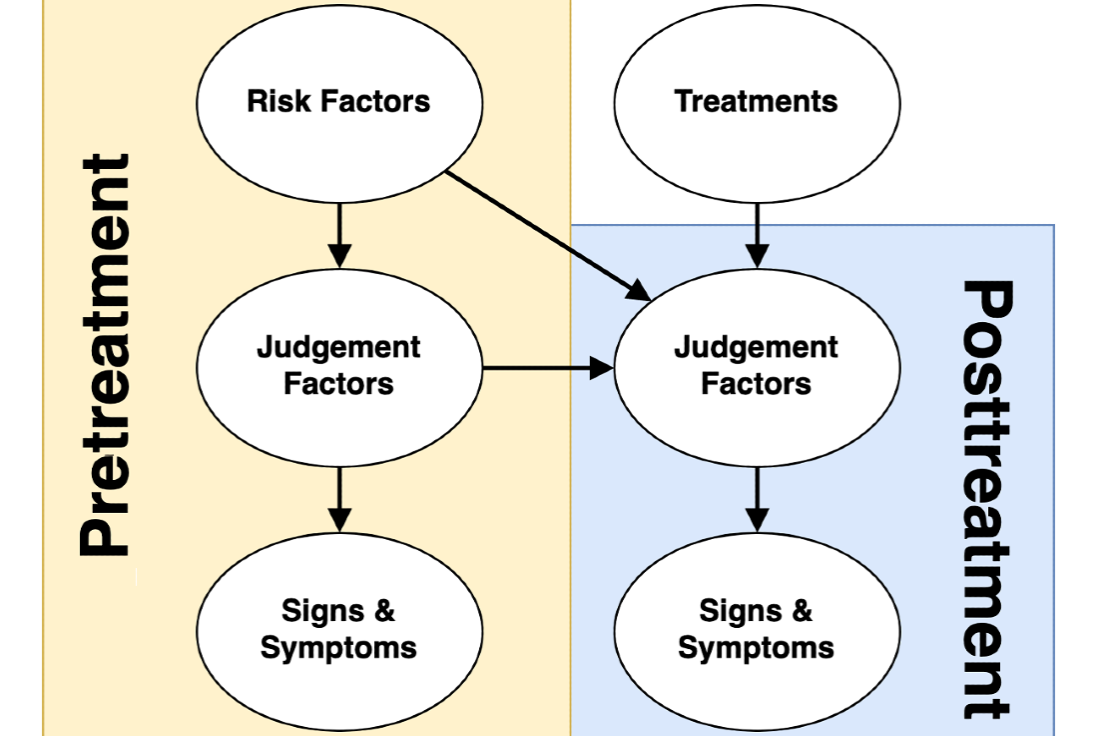
Skeleton causal diagram involving the categories of variables to be considered.

Ad hoc building of the BN causal structure is prone to biases and modeling error, which can lead to overly complex models that are not repeatable in later studies [20]. To mitigate this potential issue and to form concepts familiar to the clinical participants, we have constructed a conceptual model where potentially relevant factors in the assessment and treatment of LBP will be grouped into either “risk factors,” “signs and symptoms,” or “judgement factors.” A list of potential treatments will also be elicited. The categories have been connected in the fashion as shown in Figure 2 to form a “skeleton causal diagram,” which approximates the clinical reasoning process. This is necessary for the model functionality and makes the elicitation process recognizable for participants.

**Figure 3:**
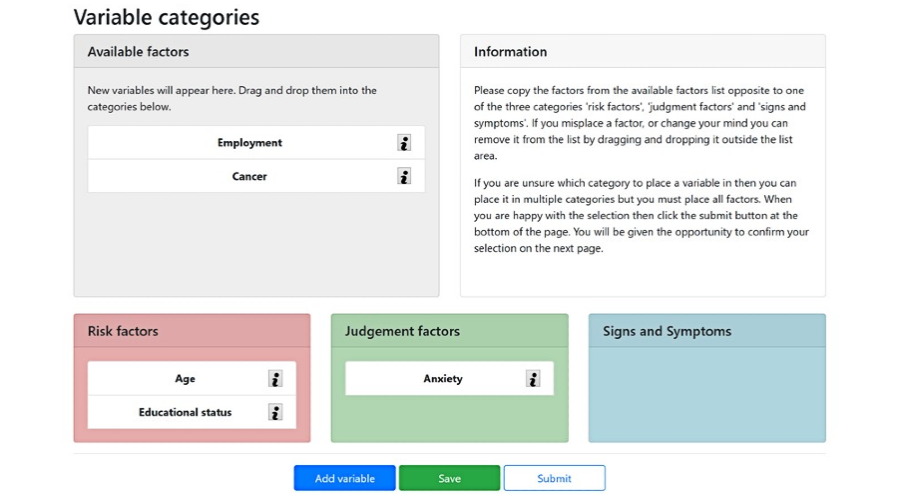
Mockup of the online interface for stage 1 of the process.

Conventional appropriateness scoring usually relies on a Likert scale [19]; however, it is anticipated that participants will have considerable time constraints and the number of potential variables will render the task unfeasible. We will therefore use a placement and ranking procedure. At the start, participants will see many example variables chosen by the research team (Figure 3) (reasons behind this decision are presented in the Results section). They can also create new variables. They will then be asked to place them into one of the three categories of their choosing and order the three associated lists so the variables they deem most important for the assessment of patients with LBP appear at the top. Unfortunately, this setup does create the risk of anchoring biases, but we hope this will be mitigated when the variables are voted on in the workshop.

To aid their understanding, participants will be given explanatory information about what constitutes risk factors, signs and symptoms, and judgement factors (Table 1), as well as many example variables from the prepopulated lists. Additionally, the meaning of each variable will be clarified by giving a question that could be answered to find out the value of the variable (such as “What is the patient’s anxiety level?”) and indication of the mode and units of measurement of the variable to ensure consistent responses.

**Table 1:** Categories of variables in the Bayesian network.

Category name^a^	Definition
Risk factor	These are pre-existing factors that might raise (or lower) the chances of the patient having a certain judgement factor or characterization of low back pain. They may also have an effect on the efficacy of the treatment but, crucially, are factors (like age and demographics) that cannot be changed by the treatment.
Signs and symptoms	These are factors that arise as a consequence of the patient having certain conditions or judgement factors. In contrast to risk factors, signs and symptoms are factors that may change or respond to treatment.
Judgement factors	These are clinical reasoning factors or the characterization of the patient’s low back pain. They are the factors that are likely to be unmeasurable with a Patient Reported Outcome Measure or test, but which help a clinician reason about prognosis or likelihood of recovery.

^a^Explanatory information about each type of variable will be made available to participants in order to inform the categorization of factors from the prepopulated lists and those created in the elicitation.

Each variable will be included in a particular category if more than 80% of participants allocate it. From those participants, we will take the median normalized rank (0 if placed at the bottom of the list and 1 at the top) to give an indication of preference for inclusion, which will be further adjusted by an interpercentile range (IPR) measure to measure consensus (more consensus will result in less adjustment of the median value). The top-placed variables will be selected for the face-to-face stage. We anticipate around 50 variables can be taken forward without overburdening participants, but we reserve some clinical judgement to decide on the exact number. Suggested variables from the online elicitation will be consolidated by the working team, and those judged by the clinical team to be duplicates will be removed.

After the online session, a face-to-face workshop will be held, in which participants will use the same tool. Misconceptions or differences in understanding will be clarified by the research team. A member of the research team will facilitate discussion of the variables that do not have consensus via use of open-ended questions. The face-to-face workshops will be video recorded to capture the discussions and enable postworkshop checking and clarification. The participants will then be given the opportunity to categorize and rank the variables for a second time, on the basis of the discussion. We anticipate that the mixture of not allowing new variables, the refined interface, and having numerous variables fixed in categories will reduce the elicitation burden while still maintaining an opportunity for group discussions, according to the conventional RAND process.

#### Stage 2: Structure Elicitation

**Figure 4:**
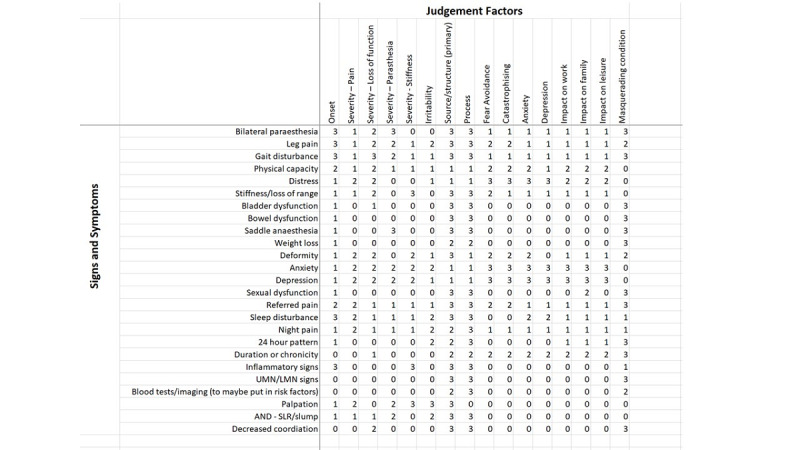
An example grid for stage 2. Relationship strengths are placed in the cells. The definitions of the scores are as follows: 0, no relationship; 1, X sometimes has a small effect on Y; 2, X sometimes has a large effect on Y or X always has a small effect on Y; and 3, X always has a large effect on Y.

The second stage seeks to connect individual variables linking risk factors to judgement factors, and judgement factors to signs and symptoms. This will be achieved by grids (Figure 4). In the online elicitation, participants will be presented with a blank grid and asked to give each relationship a strength score between 0 and 3 in the appropriate cell. The definitions of the scores are as follows: 0, no relationship; 1, X *sometimes* has a *small* effect on Y; 2, X *sometimes* has a *large* effect on Y or X *always* has a *small* effect on Y; and 3, X *always* has a *large* effect on Y, and they reflect the nuances of the BN probabilities.

We will again use the median and an 80% IPR as descriptive statistics. To make the workshop manageable (but still allow for discussion), we will fix the median score of those variables with an IPR of 1 or less (ie, the cells in the grids cannot be edited), and all others will be edited again by the participants following discussions. A subsequent overall score will be taken from the workshop for each relationship in a similar manner to the overall score in stage 1. We will then only keep the strongest relationships to form the structure of the BN, and any variables not connected to others will be discarded.

#### Stage 3: Probability Elicitation

**Figure 5:**
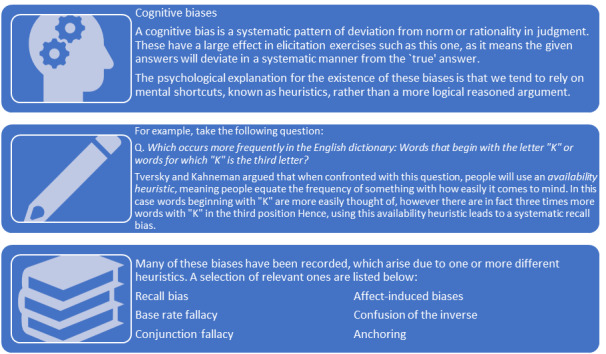
Examples of questions to be given in the cognitive bias training.

**Figure 6:**
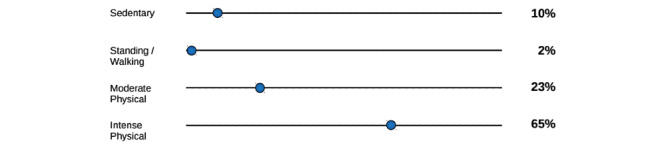
Example of direct probability elicitation for the question “What proportion of people work in the described type of job?”.

The third stage seeks to endow the BN structure with quantitative probabilities for making predictions. Crucially, although cognitive biases appear in all stages, the probability elicitation is particularly susceptible because of the quantitative nature and known issues with probability estimation [21,22]. Cognitive bias training will therefore be given prior to starting the third stage. This will include questions from unrelated fields that are susceptible to biases likely to arise within the elicitation, such as recall bias and confusion of inverse probabilities [21,22]. A brief explanation of how the bias may lead to systematic deviations in the estimates will be presented for each question (Figure 5). Moreover, we will word questions so that participants are aware of the target population and are thinking about frequency estimates, rather than individual patients (Figure 6).

The variables in the BN can be binary (eg, true and false), labeled, that is, nominal with states without any order (eg, muscle, tissue, and tendon), or ranked, that is, ordinal with states with increasing or decreasing order (eg, high, medium, and low). Additionally, some variables (eg, age) will be independent of others (known as prior variables), whereas others will be dependent (eg, the prevalence of a condition may be influenced by the age of a patient).

For binary and labeled variables, sliders will be used to allow participants to assign probabilities individually to each state. For example (Figure 6), the question “What proportion of people work in the described type of job?” has a series of nominal answers, which can be represented with individual percentages adding up to 100. In contrast, ranked variables have a smooth distribution, so participants will be asked to estimate the most likely value and the extent of the variation around that value [23].

For independent (prior) variables, a simple question similar to that in Figure 6 will suffice, whereas for conditional variables, the states of related variables will be needed, for example, “What is the probability of a patient having a fracture *given* that they have an *age* of *80+* and experienced *trauma*?”

For binary/labeled variables, the answers from the probability elicitation will be combined using logistic regression and generalized noisy OR [23] techniques to generate the probability tables for each variables. For ranked variables, we will use the answers to estimate a truncated normal distribution [24]. This reduces the number of parameters compared with other methods [25,26] and so has the advantage of avoiding overfitting, but certain nuances of the relationships are missed. Outlying answers will be identified using an IPR-based metric and removed so as not to bias the regression approach.

#### Stage 4: Validation

A subset of clinical experts will be asked to give their judgement regarding the decision-making process for a number of given scenarios, which will then be compared against the model output, otherwise known as “face validity” [27]. Further validation will likely be required to establish the efficacy of the BN in practice; however, this is outside the scope of the study.

### Internal Pilot

A much reduced version of the above protocol has already been conducted in an internal pilot study with three clinical colleagues (AH, CKJ, and DM) from Queen Mary University of London, who regularly treat patients with LBP. This was to test whether a suitable model could feasibly be constructed within a framework where the elicitation burden was not too high.

They completed all three stages of the elicitation process. During and after each stage of the process, a discussion took place regarding overall impressions of the stage in order to learn lessons and refine the process. These discussions captured clinical perspectives on the design of the questions and elicitation, feedback on what may or may not be acceptable to clinical participants, and appropriate redesign to ensure that the requirements for the modeling process were still being met. The differences in methods are discussed below, with lessons learned reserved for the subsequent Results section.

#### Stage 1: Variable Elicitation

The creation, placement, and ranking of variables were performed individually in Word documents (Microsoft Corp), with no suggested variables available from the start. Responses were collated by the computer science team and then discussed in a workshop meeting to form consensus regarding the categorization and definition of variables. Because of limited participant numbers, a final ranking was obtained via the discussion.

#### Stage 2: Structure Elicitation

This stage was performed as described in the main protocol, except for definitions of the relationship strength. Owing to limited participants, the mean and SD were used as descriptive statistics instead. A simpler definition of relationship strength (Table 1) was also used, with 0, 1, 2, and 3 simply described as “none,” “weak,” “medium,” and “strong,” respectively.

#### Stage 3: Probability Elicitation

Clinical colleagues were first asked to complete example questions based on the methods reported previously [26,28] on paper and provide their feedback. This guided the development of a primitive online tool to automatically generate the questions and record answers. Despite basic methods for easing the elicitation, the number of variables, states, and connections led to upwards of 700 questions. Completion was only feasible by splitting the questions among the three clinical members of the team and avoiding subsequent workshop discussions. Unfortunately, elicitation of probabilities associated with the treatments was abandoned owing to the volume of questions and the scope of the BN restricted to predicting judgment factors prior to treatment.

## Results

### Overview

The below text documents the outcomes of the pilot study that influenced the subsequent makeup of the described protocol. We discuss each stage separately.

#### Stage 1: Variable Elicitation

Many variables were suggested, with most having similar definitions that could be combined easily, and there was general agreement about inclusion. However, the following issues became apparent:

- Using Word documents to collate the results was cumbersome. It was also felt that the crude method could adversely affect the engagement of participants.
- There was some misunderstanding regarding the categories and how they fitted into the BN.
- There was misunderstanding and poor explanation of computer science terminology, particularly what was meant by “definition” and “states” of a variable.
- Discrepancies arose in choosing categories for certain variables. For example, “depression” could be considered a risk factor for LBP, a sign and symptom in cases where depression was a result of LBP, or a judgement factor where depression was thought to be the main driver of the patient’s condition.
- There was no formal quantitative procedure for ranking the variables.
- The time taken to discuss all the suggested variables was too long.

The use of an online interface has been influenced by the issues described. We have decided to present the participants with a number of prepopulated variables based on the pilot study together with a review of relevant literature [29-31]. This is to help them understand the terminology, allow us to gather better statistics, and reduce elicitation burden. In addition, this would highlight the difficultly in placing certain variables (eg, the “depression” example above). One of the prepopulated examples will highlight this issue to allow the participants to decide on the best handling of these variables. As described in the methods, participants will still be given the ability to add as many variables as they deem appropriate to help avoid biasing results toward the pilot.

#### Stage 2: Structure Elicitation

The process of filling in the grids to define relationships was understandable and streamlined; however, the main issue concerned the time burden, arising as a result of too many variables passed from stage 1. The following issues were also identified:

- The mean and SD measure frame consensus in terms of averaged values and so can be affected by outliers, which is not suitable for the full study.
- Similar to stage 1, the Excel files were cumbersome to manage and made preserving anonymity difficult to avoid potential biases.
- The wording of the relationships (none to strong) did not convey the nuances of the relationships between variables that could occur in the BN.

Again, the decision to use an online interface came from these issues of data collection, engagement of the participants, and anonymity of responses. For the main protocol, we have updated the definition of the relationships and introduced more appropriate statistical measures.

#### Stage 3: Probability Elicitation

The use of the online interface was, in general, a success, making the acquisition of data more streamlined and user friendly. This success, along with issues identified in previous stages of the pilot, was the reason for our decision to transfer all stages of the process online. However, beyond the sheer number of questions, we identified the following issues:

- Cognitive biases were mentioned but not adequately explained or motivated, and relevant examples were not included.
- Numerous issues with the questions and online interface were raised, including (1) eliciting the distributions required different questions depending on the variable type; (2) incorrect/strange wording of the questions; (3) questions about individual probabilities rather than frequency estimates (the former is more cognitively challenging); (4) lack of information about the related variables was not included, meaning participants had to refer elsewhere, thus slowing the process down; and (5) technical terminology displayed for ranked variables, such as “mean” and “variance,” was not very intuitive.

As mentioned in the protocol, we have introduced targeted cognitive bias training to help overcome those specific issues. Interface issues will also be addressed during development. The main issue is the elicitation burden, and the pilot has shown that any follow-up workshop would be too time consuming for participants. Therefore, instead, possible outlying answers will be identified using an IPR-based metric and removed so as not to bias the regression approach.

#### Stage 4: Informal Validation

Following the probability elicitation, a tentative BN was constructed using the AgenaRisk software [32] and compared against fictitious case study scenarios. This was in order to check whether the process was capable of returning a suitable model that would be meaningful to clinicians. The scenarios represented patients presenting with signs of serious underlying pathology, inflammatory pathology, and nonspecific LBP. The BN appeared to reason in a similar qualitative manner to that which the clinicians would expect.

## Discussion

### LBP Clinical Reasoning

We believe we have created a process that compliments the clinical reasoning process familiar to domain experts by reflecting our mathematical variables into distinct recognizable categories and asking them to describe how variables within those categories are related. Enumerating probability relationships is an area in which we anticipate experts will be least comfortable; those values are often implicitly known but almost never explicitly expressed in clinical practice. We have attempted to mitigate this by identifying common cognitive biases [21,22] and designing the elicitation to reduce the burden.

Giving this structure to the elicitation and subsequently the BN will provide confidence to future users about the “reasoning” performed by the tool. We hope the role of clinicians within the process will help alleviate concerns among users about its technical nature. Second, the structure is relatable and can be interrogated by the end user to understand the outcomes from the tool. Additionally, although the overall BN outline (Figure 2) has been designed specifically for this project, we believe it to represent clinical reasoning in a broad array of musculoskeletal conditions.

### Methodology for Eliciting Expert Opinion

There exists a wealth of literature concerning the elicitation of BNs and associated probabilities [14,15,24-28]. However, we are not aware of previous attempts to elicit comparably sized BNs with such limited time constraints on experts. To increase the chances of success, many of the individual components will use tried and tested methods, such as Noisy OR [23] and ranked node approximation [24]. Nevertheless, combining them all together in this fashion poses a major challenge.

Using the Delphi/RAND methodology [10] will help mitigate biases via group discussion and give participants an opportunity to reach a consensus. Considering an approach from the report by Ritchie et al [33], the discussion will need to remain flexible enough to explore in depth the contentious issues raised by the participants, with open questioning to avoid bias from the team that has already piloted the method. Additionally, there will likely need to be exploration of divergent views, drawing attention to elicited themes where participants have disagreed.

The very basic validation process already conducted gives encouragement that this method is viable for the purpose of eliciting a clinical reasoning BN for LBP; however, the study team recognizes the inherent bias in conducting an internal validation. As mentioned, our Delphi/RAND process has been designed to help remove such biases, but it still remains to be seen whether the process conducted with external experts will yield a clinically credible BN.

### Other Uses for the Methodology

This methodology, should it be successful in the main study, could provide a new framework for developing decision support for other musculoskeletal problems and for other complex interventions in medicine. Additionally, it could provide a learning tool for aspiring expert clinicians to confront their cognitive biases, as it did for the pilot study colleagues.

### Conclusion

We propose a protocol for developing a complex expert-driven BN decision support tool while minimizing the elicitation burden on experts, who, as professional clinicians, have limited time to offer. A basic version of the elicitation method has been internally tested with a small group of clinician researchers in a pilot study, which has yielded credible results. The proposed protocol aims to establish consensus among users by using appropriate scoring metrics and subsequent workshops to draw consensus in a Delphi-like process. This will establish the content of the model as well as the inclusion of probability values to enable scenario testing. The initial skeleton BN, derived from the internal pilot, performed well enough with simple validation to suggest that a robust BN may be achievable from implementation of the protocol.

## Abbreviations

AI: artificial intelligence
BN: Bayesian network
IPR: interpercentile range
LBP: low back pain
